# Isolation and purification of glycoglycerolipids to induce apoptosis in breast cancer cells

**DOI:** 10.1038/s41598-020-80484-x

**Published:** 2021-01-14

**Authors:** Muhammad Raisul Abedin, Sutapa Barua

**Affiliations:** Department of Chemical and Biochemical Engineering, Missouri University of Science and Technology, 110 Bertelsmeyer Hall, 1101 N. State Street, Rolla, MO 65409-1230 USA

**Keywords:** Drug delivery, Nanostructures, Chemical engineering, Breast cancer

## Abstract

Monogalactosyldiacylglycerol (MGDG) is the most abundant type of glycoglycerolipid found in the plant cell membrane and mostly in the chloroplast thylakoid membrane. The amphiphilic nature of MGDG is attractive in pharmaceutical fields for interaction with other biological molecules and hence exerting therapeutic anti-cancer, anti-viral, and anti-inflammatory activities. In this study, we investigated the therapeutic efficacy of cyanobacteria derived MGDG to inhibit breast cancer cell growth. MGDG was extracted from a cyanobacteria *Synechocystis* sp. PCC 6803 followed by a subsequent fractionation by column chromatographic technique. The purity and molecular structure of MGDG were analyzed by nuclear magnetic resonance (NMR) spectroscopy analysis. The presence of MGDG in the extracted fraction was further confirmed and quantified by high-performance liquid chromatography (HPLC). The anti-proliferation activity of the extracted MGDG molecule was tested against BT-474 and MDA-MB-231 breast cancer cell lines. The in vitro study showed that MGDG extracted from *Synechocystis* sp. PCC 6803 induced apoptosis in (70 ± 8) % of BT-474 (*p* < 0.001) and (58 ± 5) % of MDA-MB-231 cells (*p* < 0.001) using ~ 60 and 200 ng/ml of concentrations, respectively. The half-maximal inhibitory concentration, IC_50_ of MGDG extracted from *Synechocystis* sp. PCC 6803 were (27.2 ± 7.6) and (150 ± 70) ng/ml in BT-474 and MDA-MB-231 cell lines, respectively. Quantification of caspase-3/7 activity using flow cytometry showed (3.0 ± 0.4) and (2.1 ± 0.04)-fold (*p* < 0.001) higher protein expressions in the MGDG treated BT-474 and MDA-MB-231 cells, respectively than untreated controls conferring to the caspase-dependent apoptosis. The MGDG did not show any significant cytotoxic side effects in human dermal fibroblasts cells. A commercially available MGDG control did not induce any apoptotic cell death in cancer cells substantiating the potential of the MGDG extracted from *Synechocystis* sp. PCC 6803 for the treatment of breast cancer cells through the apoptosis-mediated pathway.

## Introduction

Glycoglycerolipids (GGLs) are natural products abundantly found in the cell membrane of marine algae^1,2^, cyanobacteria^3,4^, and higher plants^3–7^, with one or two carbohydrate units, glycerol, and diversified acyl lipid groups^7,8^. GGLs are conserved in both gram-negative and gram-positive bacteria, *i.e.*, *Bacillus pumilus*^9^, *Lactobacillus plantarum*^10^, *Microbacterium* sp.^11^, *Micrococcus luteus*^12^, and *Phormidium tenue*^13^ with highly conserved structures. The amphiphilic nature of GGLs is attractive in pharmaceutical fields for interaction with other biological molecules and hence exerting therapeutic anti-cancer^14–16^, anti-viral^17,18^, and anti-inflammatory activities^19,20^. GGLs potently inhibit angiogenesis^21^, cancer cells^14^, and solid tumor^16,22,23^ growth by selectively inhibiting the replicative DNA polymerase activity both in vitro and in vivo^15,16,23^. Monogalactosyl diacylglycerol (MGDG), a GGL isolated from spinach, combined with gemcitabine anti-cancer drug revealed synergistic effects of inhibitive proliferation on human pancreatic cancer cell lines BxPC-3, MiaPaCa2, and PANC-1 through the inhibition of DNA replicative pols alpha and gamma activities, compared with MGDG or gemcitabine alone^24^. The fractions of GGLs e.g. MGDG, digalactosyl diacylglycerol (DGDG), and sulfoquinovosyl diacylglycerol (SQDG) in spinach potently affect in vitro colon cancer cells, angiogenesis, and solid tumor growth via their inhibitory activities of DNA polymerase^16,21–23^. Naturally occurring sulfoquinovosylglycerolipids show promising anti-proliferative activity toward human cancer cells not only by targeting DNA polymerases^25^, but also inhibiting the mitotic centromere-associated kinesin (MCAK)^15^. Another interesting feature of these compounds is their involvement in cell recognition and signaling which make them promising agents in drug delivery systems^26^. Several GGLs have been related to the activation of natural killer T cells, which is a central event in a variety of immune responses including the development of autoimmunity, tolerance, and maintenance in defense responses to tumors and infectious agents^27^. A series of sulfonic acid-containing GGLs isolated from cultured cyanobacteria (blue-green algae) have been discovered as a new class of compounds to inhibit the cytopathic effects of the human immunodeficiency virus (HIV-1)^18^. The high potential of GGL therapy strategy has attracted great interest in recent years and thus is explored to evaluate its therapeutic efficiency for the treatment of breast cancer cells.

We investigated the anti-proliferative efficacy of marine cyanobacterium *Synechocystis* sp. derived MGDG molecule on human epidermal growth factor receptor-2 (HER2)-positive BT-474, MDA-MB-231 triple-negative breast cancer cells, and human dermal fibroblasts. MGDG molecule is more abundantly and predominantly present in cyanobacterial cell chloroplast membrane than in plant sources. MGDG constitutes nearly 60% of the chloroplast lipid in *Synechocystis* sp. cyanobacterial cells. The marine cyanobacteria *Synechocystis* sp. derived MGDG molecule predominantly contains 18:3 n-6 GLA at the sn-1 fatty acid chain. *Synechocystis* sp. PCC 6803 was chosen because monogalactosyldiacylglycerol (MGDG) from this strain contains 18:3 n-6 GLA at the sn-1 fatty acid residues making it a more lipophilic molecule than other polar MGDG molecules with 16:0 carbon fatty acid residues^28^. This characteristic of glycoglycerolipids from *Synechocystis* sp. PCC 6803 would catalyze the intracellular uptake and high biological activation to induce apoptosis in breast cancer cells. Polyunsaturated fatty acids containing 18:3 γ- linolenic acid (GLA) showed higher apoptosis in various cancer cells (cervical, gastric, and prostate cancers) than normal cells both in vitro and in vivo by generating reactive oxygen species, suppressing angiogenesis via Akt cell signaling pathway and inducing apoptosis^29–32^. The n-6 GLA molecule has been shown to sensitize breast cancer cells to antimitotic and endocrine therapeutic drugs in vitro. The n-6 GLA molecule, a member of the ω-6 fatty acids selectively inhibits breast tumor cells without affecting the healthy cells^33,34^. Hence, the *Synechocystis* sp. PCC 6803 cyanobacteria derived MGDG molecule is of great interest in the current study. We isolated MGDG with high purity from the total lipid extract of *Synechocystis* sp*.* PCC 6803 cyanobacterial cells. In this study, we comparatively investigated the anti-proliferative and apoptosis induction efficacy of the cyanobacterial extract MGDG with high n-6 GLA content and plant extract n-3 ALA-rich pure MGDG in BT-474 and MDA-MB-231 breast cancer cells.

## Materials and methods

### Growth kinetics of *Synechocystis* sp. PCC 6803

*Synechocystis* sp. PCC 6803 was a kind donation from Dr. Himadri Pakrasi’s laboratory at Washington University in St. Louis^7,35–38^. The *Synechocystis* sp. was cultured in batch cultures of 10 ml solid and 250 ml liquid BG11 media at 30 °C and 300 rpm in a rotary shaker under 30 μmol/m^2^/s (~ 2200 lx) fluorescent lamps^39^. Fluorescence microscopic images of PCC 6803 and the corresponding spectra analysis were captured using a scanning laser inverted confocal microscope (Ti-Eclipse; Nikon). For the growth curve analysis, the species was inoculated in a 5 ml liquid BG11 media. 150 μl aliquots of liquid samples were transferred to a 96 well plate (Corning) at different times to measure the growth kinetics of PCC 6803 after adjusting the initial absorbance at 680 nm, $A_{680,0}$ = 0.05 at 24 h. BG11 media alone without any inoculum was used as a negative control. The maximum absorbance at the wavelength of 680 nm was measured using a microplate reader (BioTek). The specific growth rate, $\alpha$ was computed using Eq. (3).

1 $$ \frac{{dA_{680} }}{dt} = \alpha N $$

where $A_{680}$ is the absorbance of cells at time, $t$. By integrating Eq. (1) from $t = 0$ to $t = t$,

2 $$ ln\frac{{A_{680t} }}{{A_{6800} }} = \alpha \left( {t - t_{0} } \right) $$

3 $$ \alpha = \frac{{ln\frac{{A_{680t} }}{{A_{6800} }}}}{{\left( {t - t_{0} } \right)}} $$

The doubling time, $t_{d}$ is calculated using Eq. (4):

4 $$ t_{d} = \frac{0.693}{\alpha } $$

### Total lipid extraction from *Synechocystis* Sp. PCC 6803

The total lipid was extracted from the *Synechocystis* sp. using the Bligh and Dyer Method^40^. Cell pellets from 250 ml of culture were collected at $A_{680,t}$ = 3.0 after 120 h, centrifuged at 15,000 g for 15 min and suspended in deionized water for two times to remove residual medium. One ml of cell suspension was mixed with 3.75 ml of chloroform: methanol (1:2, v/v) solution for 15 min using a vortex mixer. After thorough mixing, 1.25 ml of chloroform was added and vortexed for 1 min. Two distinctive layers were obtained after adding 1.25 ml of water to the mixture. The chloroform layer (lower phase) contained all the lipids, and the aqueous methanol layer (upper phase) contained the non-lipid fractions. The upper phase was discarded carefully, and the lower phase was collected. Chloroform was then evaporated completely under vacuum in a fume hood to obtain the total lipid. Total lipid was reconstituted in 1 ml of chloroform and stored in −20 °C.

### Isolation of MGDG using column chromatography

Total lipid was fractionated using the normal phase column chromatography technique. A manually packed column was used for the isolation process (SI Fig. 1a). Silica gel (particle size 10–40 microns) was used as a stationary phase. Chloroform and acetone were used as mobile phases. A gradient elution method was applied for the fractionation (SI Table 1). Samples (A-K) were collected for each combination of the eluent and labeled for further detection, structural analysis, and quantification (SI Fig. 1b).

### Identification of MGDG using NMR analysis

The presence of the MGDG molecule in the eluted fractions was investigated using ^1^H NMR analysis. Each of the eluted fractions was analyzed to investigate the presence of MGDG molecule. The plant extract standard MGDG molecule was used as a reference. MGDG (> 99% purity, MW: 752.369) was purchased from Avanti Polar Lipids Inc. Chloroform-d (CDCl_3_) was chosen both as the reference and the solvent for NMR analysis. The NMR peaks have been interpreted from the lipid handbook NMR database^41^ and the previous studies on structural analysis of plant-derived GGLs^4,42^.

### Quantitative analysis of MGDG using HPLC–UV

The extracted MGDG molecule was both detected and quantified using a HPLC–UV system. The following instruments and solvents were used for chromatographic analysis. Column: LiChrospher 100 Diol (particle size: 5 µm, column size: 250 × 4 mm) (Millipore Sigma), guard column: LiChroCart 4–4, mobile phase: chloroform (solvent A), methanol–water (95:5) (solvent B), detector: UV, injection volume: 20–25 µL, flow rate: 1 ml/min. The MGDG molecule was detected by the UV detector at 240 nm. The gradient elution method used in HPLC is shown in SI Tables 2 and 3^43^. The peak areas corresponding to the known concentrations of standard MGDG are shown in SI Table 4.

### In vitro therapeutic efficacy

BT-474, MDA-MB-231, and human dermal fibroblast cells (ATCC) were cultured in Hybri-care ATCC 46-X medium, RPMI 1640, and fibroblast growth factor supplemented basal medium (ATCC), respectively, supplemented with 10% fetal bovine serum (FBS) and 1% penicillin–streptomycin at 37 °C and 5% CO_2_. Approximately, 10,000 cells per well were plated in 96-well plates and treated with 0–200 ng/ml doses of standard MGDG and isolated from *Synechocystis* sp. by column chromatography technique. To examine the cytotoxicity effects on human dermal fibroblasts, the cells were treated with 100 ng/ml of MGDG extracted from *Synechocystis* sp. and MGDG standard. After 72 h of incubation, cell viability was assessed using MTT (3-(4, 5-dimethylthiazol-2-yl)-2, 5-diphenyltetrazolium bromide, MW 414) assay. MTT reagent was added to each well to convert into an insoluble formazan from water-soluble MTT. After 4 h, sodium dodecyl sulfate (SDS) solution prepared in 0.01 N HCl was added to each well to solubilize the formazan. The percentage of live cells proportional to the absorbance value of solubilized formazan solution was quantified by measuring the absorbance at 570 nm (BioTek Synergy 2). The absorbance value was adjusted by subtracting the mean absorbance level of wells containing medium only. Cell viability was calculated as a means of six wells containing BT-474, MDA-MB-231, and dermal fibroblast cells. The cells treated with PBS were used as live control that was placed in every alternative column in the well plate adjacent to corresponding dosage or treatments to remove the variability in the number of seeded cells in adjacent wells. The live controls were replicated at least six times for each dosage. For dead control, we treated the cells with saponin in at least six replicates (SI Fig. 2). The % cell viability was calculated as follows Eq. (5):

5 $$ \% cell\,viability = \frac{{A_{570\,of\,sample} - A_{570\,of\,medium } }}{{A_{570\,of\,live\,cells} - A_{570\,of\,medium } }} \times 100 $$

### Apoptosis assay

The BT-474 and MDA-MB-231 cells were investigated for the caspase-dependent apoptosis mechanism by staining for the cleaved or active form of caspase-3/7 marker fluorescence dye. The cells at a density of 10,000 cells/ml were seeded in 96 well plates in replicates of at least n = 3. The cells were treated with MGDG extracted from Synechocystis sp. PCC 6803 and MGDG standard and were incubated for 48 and 72 h. The cells treated with PBS were used as live control with at least three replicates. After incubation, cells were stained with green fluorescent active caspase-3/7 marker (Invitrogen). The cells were imaged in the phase contrast field along with green fluorescent signals using a Zeiss Apotome 2.0 microscope equipped with a 10X objective, 470/40 excitation filter, and 525/50 nm emission filter. To quantify the caspase-3/7 protein expression, 50,000 each of BT-474 and MDA-MB-231 cells were treated with 60 and 100 ng/ml of MGDG, respectively. Cells treated with 10 μl of PBS was used as untreated control. After 48 and 72 h of incubation, cells were washed twice using 100 μl of PBS and stained with 5 μΜ of caspase-3/7 green fluorescence apoptosis marker in 100 μl of the respective medium. The cells were incubated for 30–45 min at 37 °C. The fluorescence data was detected for 20,000 events/sample and acquired using a flow cytometer (BD Accuri C6 plus). The bandpass filters used in the flow cytometry were 530/30 and 585/40, respectively. The laser excitation wavelength was 488 nm.

### Statistical analysis

Each experiment was carried out in independent repetitions to have at least triplicates valid measurements. The results were reported as mean ± standard deviation and analyzed using the Student’s t-tests with two-tailed hypotheses and using the JMP statistical software (version 15, SAS Institute). *p* < 0.001 was considered as statistically significant and was denoted by ***.

## Results

### *Synechocystis* sp. growth curve kinetics and molecular structure of MGDG

**Figure 1:**
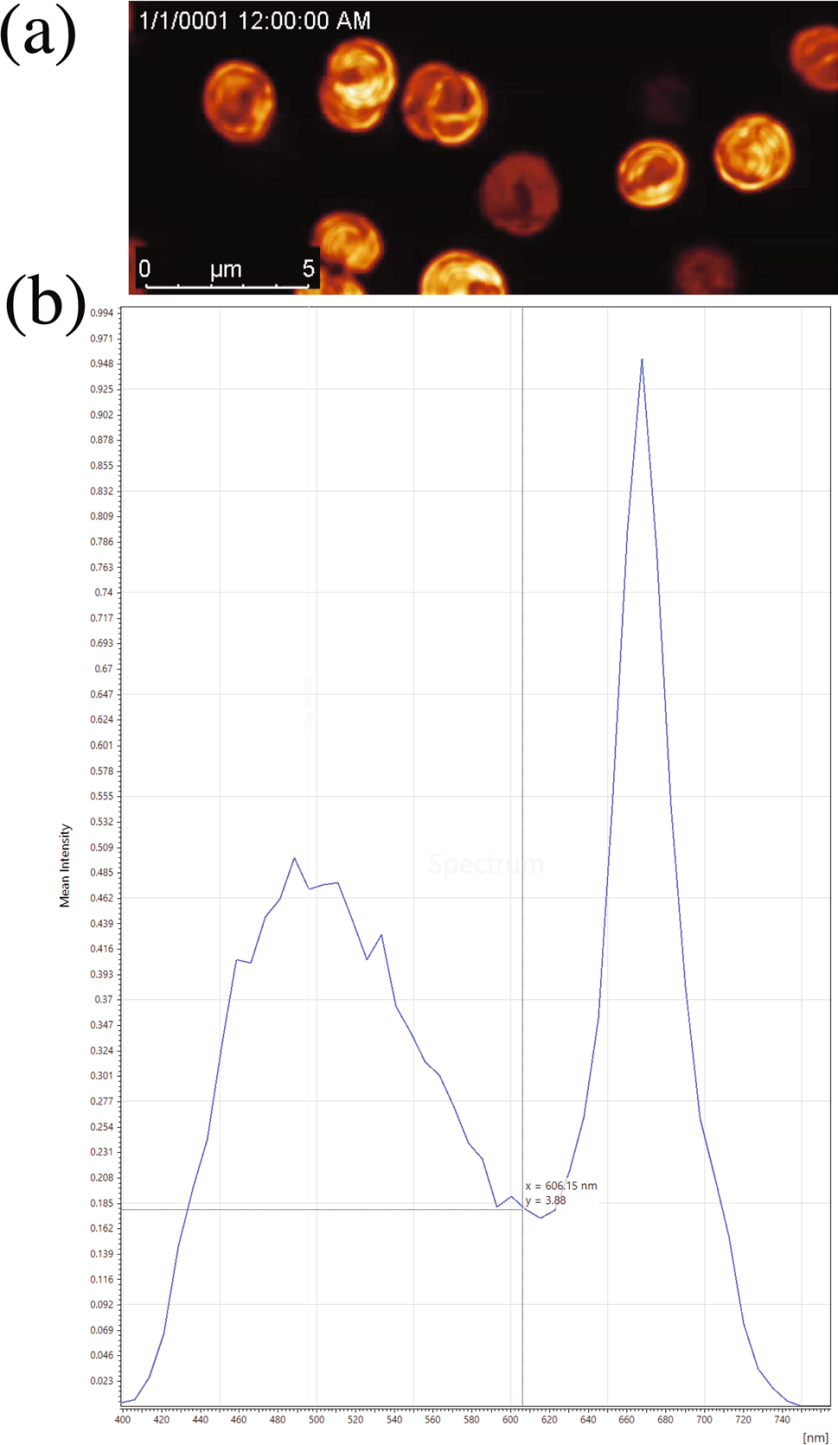
(**a**) Time-lapse video microscopic images of *Synechocystis* sp*.* PCC 6803, and (**b**) its fluorescence emission spectra analysis. Red color indicates the abundance of photosynthetic pigments.

**Figure 2:**
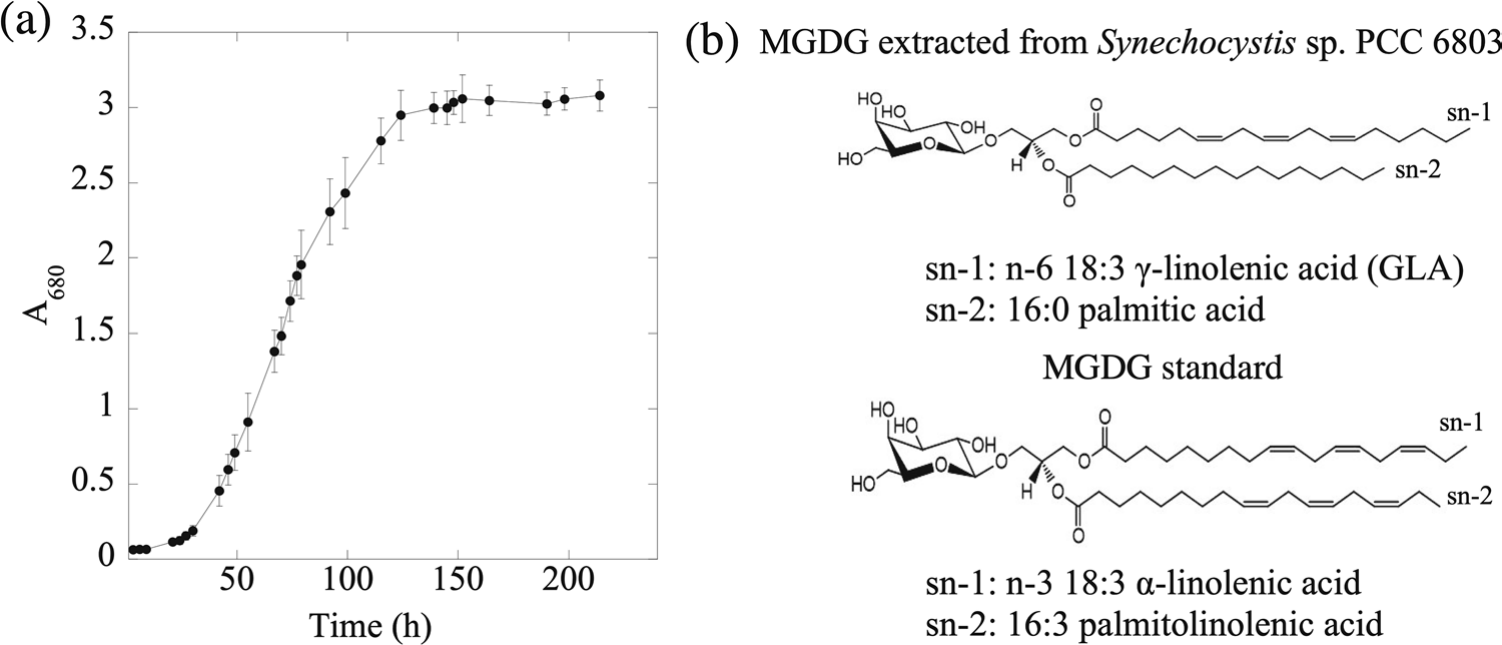
The growth kinetics of *Synechocystis* sp. and structure of MGDG. (**a**) Growth curve of *Synechocystis* sp. PCC 6803 wild type strain. The growth was measured in terms of absorbance value obtained at 680 nm. (**b**) Structure of MGDG extracted from *Synechocystis* sp. PCC 6803 containing n-6 18:3 γ-linolenic acid (n-6 GLA) and 16:0 palmitic acid at sn-1 and sn-2 fatty acid chains, respectively (upper panel); the most abundant structure of plant extracted MGDG standard containing n-3 18:3 α-linolenic acid (n-3 ALA) and 16:3 palmitolinolenic acid at sn-1 and sn-2 fatty acid chains, respectively (lower panel).

Figure 1 demonstrates the confocal fluorescence images and fluorescence emission spectra analysis of *Synechocystis* sp. PCC 6803, indicating that the microorganism grew properly with the highest absorbance spectrum at 680 nm^44,45^. The growth kinetics of *Synechocystis* sp. PCC 6803 was studied to obtain the maximum total lipid yield. The growth curve shows that the lag phase extended for almost 30 h (Fig. 2a). It took the species for six days to reach the stationary phase. The specific growth rate, $\alpha$ was calculated as 0.04 h^−1^. The doubling time ($t_{d}$) was calculated as 18.3 h. We extracted the MGDG molecule from the cells at the beginning of the stationary phase for maximum yield. Structurally, the MGDG molecule is constructed on a glycerol backbone (Fig. 2b). The galactose residue is bound to the sn-3 position of the glycerol backbone via a β-anomeric linkage. This head group remains conserved across the average molecular structures found in plant and cyanobacterial sources. The molecule has two long fatty acid chains in the sn-1 and sn-2 position of the backbone. The long fatty acid chains show variability in length, double bond position, and degree of unsaturation across and within the sources^6,7^. Typically, 18:1 or 18:3 /16:3 or 16:0 (sn-1/sn-2) average fatty acid composition is common in plant extract MGDG^42,46^. In contrast, the fatty acid composition can vary from long-chain composition of 18/16 (sn-1/sn-2) to a very long chain composition of 34 configurations in *Synechocystis* sp. extract MGDG^6,47^. Mostly, the *Synechocystis* sp. PCC 6803 contains the 18:3 GLA polyunsaturated fatty acid chain at the sn-1 position and 16:0 palmitic acid saturated fatty acid chain at the sn-2 position^6,48,49^.

### NMR analysis confirms the isolation of MGDG from total lipid extract

**Figure 3:**
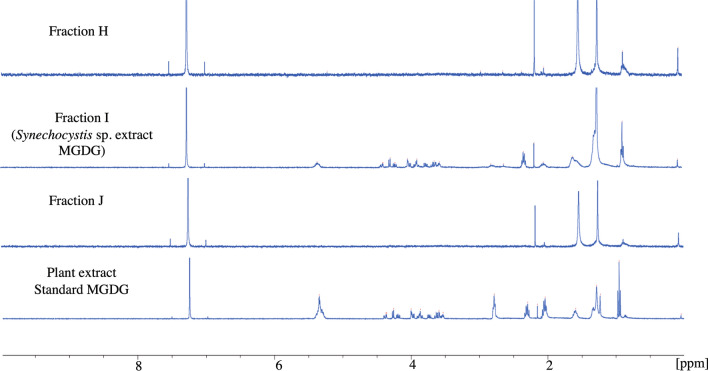
Confirmation of the presence of MGDG extracted from *Synechocystis* sp. PCC 6803 in fraction I. ^1^H NMR analysis of *Synechocystis* sp. extracted fractions H, I, and J with 40% chloroform and 60% acetone eluent combination. The NMR spectra of the MGDG standard are shown in the lowest panel. The NMR analysis confirms the presence of MGDG molecule in fraction I.

**Figure 4:**
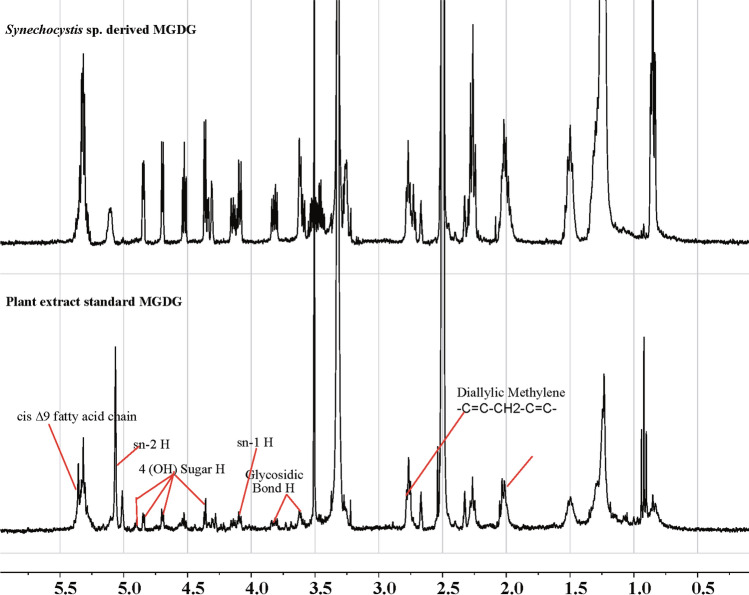
Structural identification of MGDG extracted from *Synechocystis* sp. PCC 6803. ^1^H NMR analysis of MGDG extracted from the *Synechocystis* sp. shows that the fraction molecule (upper panel) contains all the major structural H components δ 2.8 (diallylic methylene), δ 3.6 and 3.8 (Glycosidic H moiety), δ 4.18 (sn-1 H), δ 5.15 (sn-2 H), δ 5.25 cis ∆9 moieties present in the standard MGDG molecule (lower panel). The cyanobacterial NMR spectra are identical to the NMR spectra of the MGDG standard confirming the purity of the extracted MGDG molecule.

MGDG molecule was isolated from the total lipid extract of *Synechocystis* sp. PCC 6803 using column chromatography. The fractions with each eluent were collected separately and analyzed individually by NMR analysis. The MGDG molecule was isolated with an eluent combination of 40% chloroform and 60% acetone. The fractions (H, I, J) were collected with the same eluent combination. The NMR analysis confirms the presence of MGDG molecule in fraction I (Fig. 3). The set of NMR peaks of fraction I were completely identical to the standard MGDG molecule suggesting the isolation of MGDG molecule with high purity. The structure of MGDG in fraction I was analyzed from the peaks obtained in the NMR analysis (Fig. 4). The chemical shift values at δ 4.18, 5.15 represent the sn-1 and sn-2 H atom, respectively. The peaks at δ 4.29, 4.55, 4.70, and 4.85 confirm four H atoms of the OH group in the carbohydrate sugar present in MGDG. Glycosidic bond in MGDG is represented by the chemical shift δ 3.6 and 3.8. A very broad and sharp peak at chemical shift δ 5.25 might represent the unsaturated cis Δ9 fatty acid attached to the sn-1 or sn-2 position^41^. The presence of the unsaturated fatty acids can be confirmed by the diallylic methylene functional group at chemical shift δ 2.8. The results are consistent with the NMR data of MGDG reported earlier by Marcolongo *et. al.* and Maeda et. al.^4,42^.

### Detection and quantification of MGDG using HPLC–UV system

**Figure 5:**
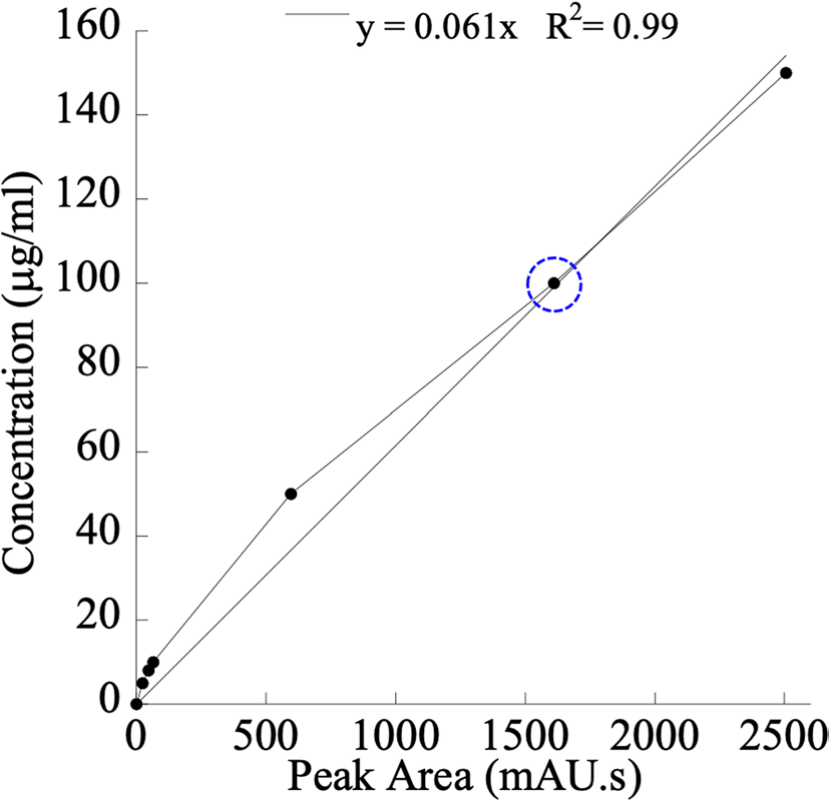
Calibration curve for quantification of MGDG extracted from *Synechocystis* sp. PCC 6803. A standard calibration curve was prepared using known concentrations of plant extract MGDG molecule and the corresponding peak areas in terms of milli-absorbance unit × seconds (mAu.s) in HPLC–UV analysis. The equation obtained for the unknown concentration of the MGDG molecule for any known peak area is y = 0.061 × with the R^2^ value of 0.99.

**Figure 6:**
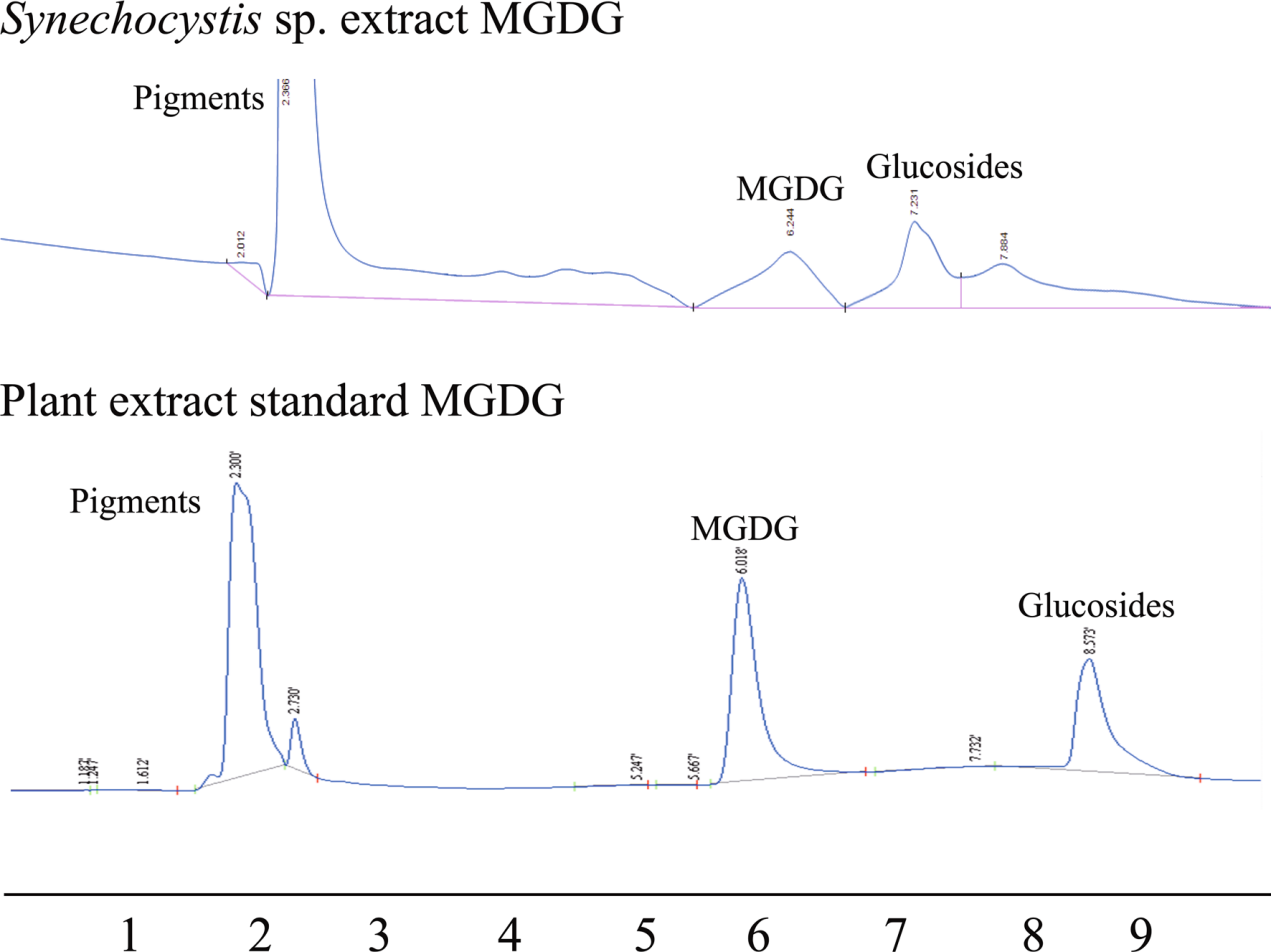
HPLC–UV analysis of MGDG extracted from *Synechocystis* sp. PCC 6803 (upper panel) and MGDG standard (lower panel). The MGDG peak was obtained at ~ 6 min for the MGDG molecule from both of the sources. The peaks obtained at ~ 2 and ~ 7–8 min represent the pigments and glucosides peaks eluted with the methanol/ water solvents.

The *Synechocystis* sp. extracted MGDG molecule was detected and quantified using the HPLC coupled with a UV detector. The correlation of area under the peak with known concentrations of standard MGDG was observed in HPLC analysis (Fig. 5). The R^2^ value of the fitted curve was 0.99. After the isolation of MGDG from total lipid extract in chromatographic fraction I, the fraction was run through the HPLC column for quantification. The peak obtained at 6.24 min confirmed the presence of the MGDG molecule (Fig. 6). The amount of detected MGDG in fraction I was quantified using the concentration correlation plot obtained in Fig. 5. Fraction I was collected at the chromatogram area of 1545.4 mAu.s as shown by the dotted blue circle in Fig. 5 to calculate the concentration of extracted MGDG in Fraction I as 94.4 μg/ml using the linear correlation: y = 0.061 x, where x and y represented the chromatographic peak area and MGDG concentration, respectively. Therefore, the mass of MGDG isolated in 200 μl of elution buffer was ~ 18.8 μg.

### In vitro anti-proliferation efficacy

**Figure 7:**
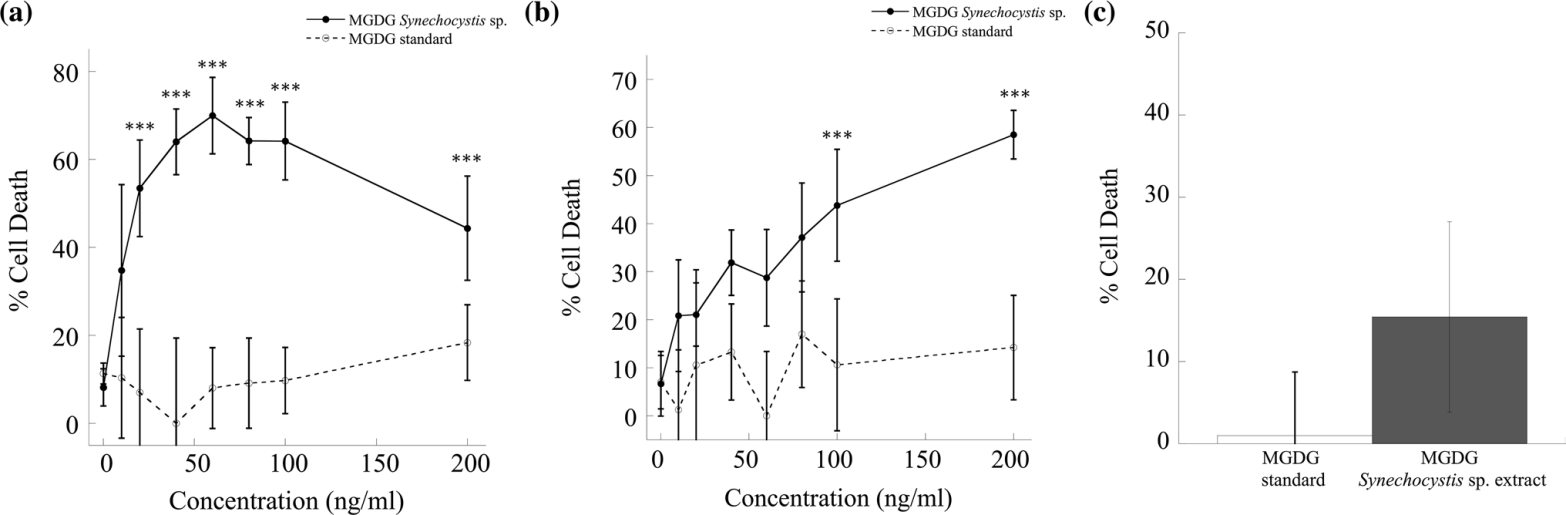
In vitro anti-cell proliferation analysis of MGDG extracted from *Synechocystis* sp. PCC 6803 (solid line) and MGDG standard (dotted line) in (**a**) BT-474 and (**b**) MDA-MB-231 cell lines. Mean percentage values are presented from six replicates (n = 6) for each drug dosage. The MGDG from the *Synechocystis* sp. inhibits up to 70% (*p* < 0.001) and 60% (*p* < 0.001) of BT-474 and MDA-MB-231 cancer cells, respectively where MGDG standard does not show any toxicity within the concentration range of 0–200 ng/ml. *** indicates the *p* value < 0.001 showing a statistically significant difference between % cell death in MGDG extracted from the *Synechocystis* sp. and MGDG standard. (**c**) MGDG extracted from the *Synechocystis* sp. does not show any cytotoxic side effects in human dermal fibroblasts normal cell line control.

To determine the anti-proliferation efficacy in vitro, the dose–response cytotoxicity of MGDG extracted *Synechocystis* sp. and MGDG standard was evaluated. The anti-proliferation activity of extracted MGDG was compared against the MGDG standard in HER2-positive BT-474 (Fig. 7a) and MDA-MB-231 triple-negative breast cancer cells (Fig. 7b). In BT-474 cells, MGDG extracted from *Synechocystis* sp. inhibited up to 70% cell proliferation over the concentration range of 0–200 ng/ml (•, Fig. 7a). The maximum inhibition in BT-474 cells was (70 ± 8) % at 60 ng/ml (*p* < 0.001). At higher concentrations (> 60 ng/ml), a decrease in % cell death was observed in BT-474 cells indicating cell-dependent drug resistance to MGDG extracted from *Synechocystis* sp^50,51^. In MDA-MB-231 cell line, for the concentration range of 0–200 ng/ml, MGDG extracted from *Synechocystis* sp. showed significant cell growth inhibition from 0 to 60% in a dose-dependent manner (•, Fig. 7b). The extracted MGDG inhibited (58 ± 5) % of MDA-MB-231 breast cancer cells at the concentration of 200 ng/ml (*p* < 0.001). The half-maximal inhibitory concentration, IC_50_ values of MGDG extracted from *Synechocystis* sp. are (27.2 ± 7.6) and (150 ± 70) ng/ml in BT-474 and MDA-MB-231 cells, respectively. In contrast, in both BT-474 and MDA-MB-231 cell lines, standard MGDG did not show any statistically significant anti-proliferation activity (< 10% cell death) compared to untreated positive control within the concentration range of 0–200 ng/ml (ο, Fig. 7a,b). Although MGDG from *Synechocystis* sp. induced the % cell death of breast cancer cells in a concentration-dependent manner, the cytotoxicity of fibroblast cells (normal cell control) was less (< 15%) than breast cancer cells after exposure to 100 ng/ml of MGDG for 72 h (Fig. 7c), indicating that breast cancer cells were more sensitive to the MGDG derived from *Synechocystis* sp. than normal cells. Taken together, the results revealed that MGDG extracted from *Synechocystis* sp. induced selective apoptotic cell death in BT-474 and MDA-MB-231 breast cancer cells with different potency after 72 h of incubation, while causing less toxicity in normal fibroblasts.

### *Synechocystis* sp. extracted MGDG induces caspase-dependent apoptosis

**Figure 8:**
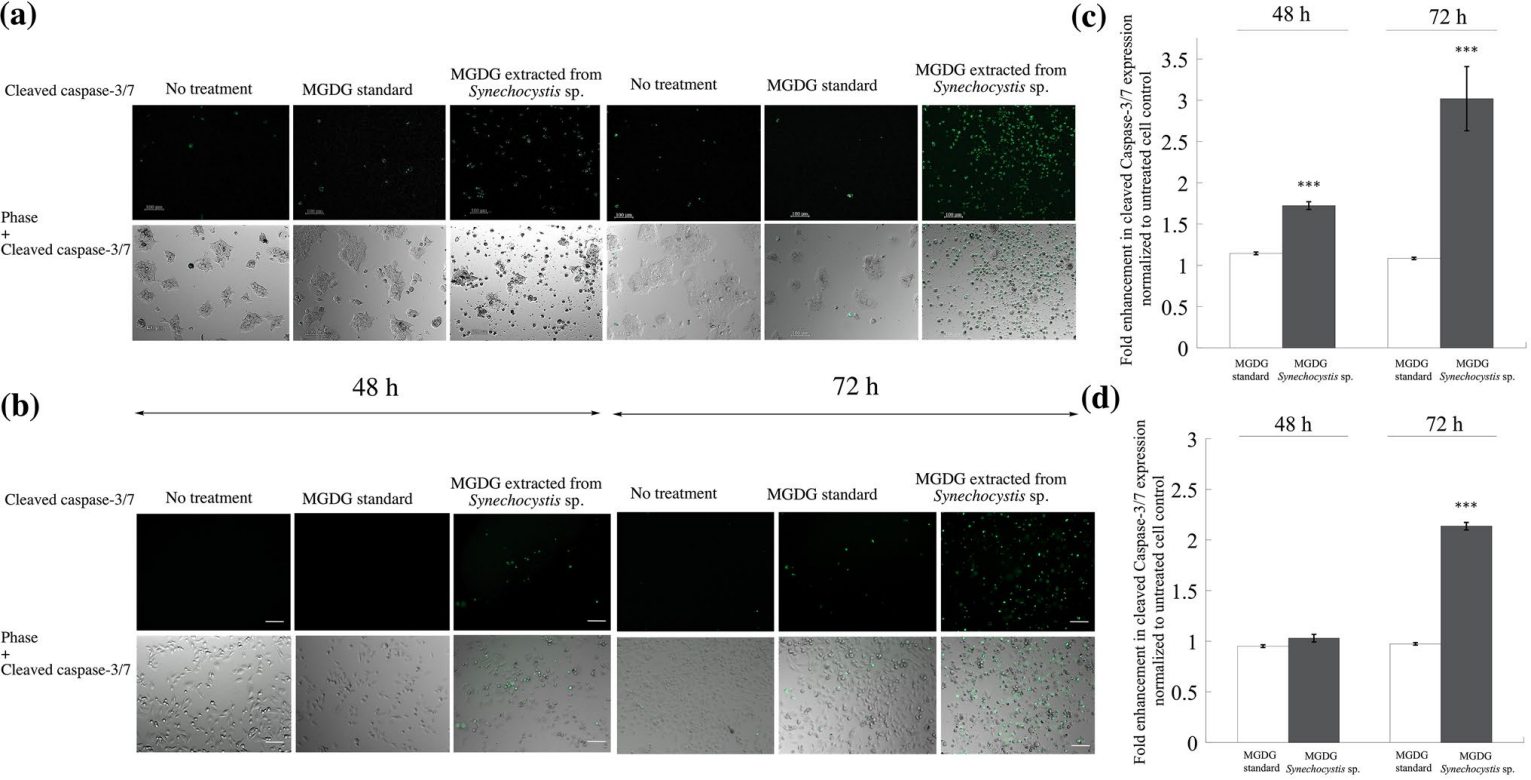
In vitro apoptosis induction by MGDG extracted from the *Synechocystis* sp. and MGDG standard after 48 and 72 h treatments in (**a**) BT-474, and (**b**) MDA-MB-231 cells. The green fluorescence of apoptotic caspase-3/7 proteins increases in both BT-474 and MDA-MB-231 cells treated with MGDG extracted from the *Synechocystis* sp. The combined phase contrast and the fluorescence images show that the cyanobacterial extracted MGDG enhances the intensity and density of green fluorescence cells indicative of caspase-dependent apoptosis in both cell lines. In contrast, cells treated with MGDG standard molecule did not show any sign of apoptosis in either cell line even after 72 h of treatment. The scale bars represent 100 μm. Quantification of the active caspase-3/7 expression in (**c**) BT-474, and (**d**) MDA-MB-231 cells treated with MGDG extracted from the *Synechocystis* sp. and MGDG standard after 48 h and 72 h of treatments. In BT-474 cells, the active caspase-3/7 expression was increased by 1.7 (*p* < 0.001) and 3 (*p* < 0.001) fold after 48 h and 72 h treatments, respectively compared to untreated control. The increase in caspase-3/7 expression was 2.1 (*p* < 0.001) fold after 72 h treatment in MDA-MB-231 cells compared to the untreated control. Mean ± standard deviation from two independent experiments in at least three replicates (n = 6) are presented. *** indicates the *p* value < 0.001, showing a statistically significant difference compared to untreated control cells.

To investigate the late phase apoptosis, BT-474 and MDA-MB-231 cells were treated with 100 ng/ml of MGDG extracted from *Synechocystis* sp. and MGDG standard and stained with green fluorescently labeled caspase-3/7, a well-known apoptotic marker. The assay utilizes a nucleic acid binding dye conjugated with a four amino acid peptide, DEVD (Asp-Glu-Val-Asp motifs) that is recognized by caspase-3/7 and cleaved, enabling the dye to bind with DNA and fluoresce at ~ 530 nm^52–54^. The conjugated dye is non-fluorescent until the DEVD peptide is cleaved by active caspase-3/7. After 48 h and 72 h of treatment, cells treated with MGDG extracted from *Synechocystis* sp. showed green fluorescently stained DNA indicating the activation of caspase-3/7 in BT-474 (Fig. 8a) and MDA-MB-231 cells (Fig. 8b). The combined phase and fluorescent signal images show the overlap of caspase-3/7 positive cells and the unhealthy shrinking cells suggesting the initiation of apoptosis. The induction of apoptosis was assessed by Western blot technique (SI Figs. 3 and 4). The Western blot analysis showed that the full-length caspase-3 (MW: 32 KDa) expression was reduced in BT-474 and MDA-MB-231 cells treated with MGDG from *Synechocystis* sp., while the protein expression did not change in cells treated with MGDG standard relative to untreated cell controls. The reduced expression of full-length caspase-3 suggests the cleavage of the protein into an active form of low molecular weight (17 kDa) active cleaved caspase-3. The cleaved caspase-3/7 expression was quantified using flow cytometry. The fold enhancement of caspase-3/7 was quantified using flow cytometry analysis (Fig. 8c,d). We observed a statistically significant increase of 1.7 ± 0.04 and 3.0 ± 0.4 (*p* < 0.001)-fold higher caspase-3/7 concentrations after 48 and 72 h, respectively in BT-474 cells treated with MGDG extracted from *Synechocystis* sp. than untreated cell controls (filled column, Fig. 8c, SI Fig. 5a,5b). The number of caspase-3/7 concentrations in MDA-MB-231 cells treated with MGDG from *Synechocystis* sp. increased significantly up to 2.1 ± 0.03-fold (*p* < 0.001) after 72 h compared to untreated cell controls (filled column, Fig. 8d, SI Fig. 5c,d). MGDG standard did not show any significant changes in cleaved caspase-3/7 expressions compared to untreated control cells. These results are in good agreement with fluorescent microscopic images indicating the cells being shrunk and round shaped with a complete halt in growth of cancer cells. In contrast, after 48 and 72 h of treatment, there was no sign of apoptosis in the standard MGDG treated compared to untreated cell controls (open column, Fig. 8c,d). The untreated cells and the cells treated with standard MGDG were compact multilayered colonized and elongated in shape in the case of BT-474 and MDA-MB-231 cell lines, respectively, and were observed healthy suggesting continuous growth capability.

## Discussion

MGDG contains fatty acyl groups derived from two fatty acid molecules at sn-1 and sn-2 position and a polar head at sn-3 position in a 3-carbon glycerol scaffold (Fig. 2b). Fatty acid molecules such as n-6 GLA may exert anti-proliferative effect by regulating genes and proteins involved in cell cycle and apoptosis, altering the cellular composition of fatty acids, and by producing downstream anti-proliferative metabolites such as 1-series prostaglandins and free radical molecules from cyclooxygenase (COX) catalyzed lipid peroxidation^55,56^. The potent anti-proliferative activity of the MGDG is mostly attributed to the fatty acyl components of the MGDG molecular structure^55,57^. The structure of the MGDG molecule and the fatty acid content of MGDG derived from *Synechocystis* sp. PCC 6803 is well studied. The molecule is rich in 18:3 n-6 GLA fatty acids. The fast atom bombardment tandem mass spectrometry (FAB-MS) analysis of glycolipids derived from *Synechocystis* sp. PCC 6803 showed 18:3 n-6 GLA, 18:2, and 18:1 combination of fatty acid chains with a relative abundance of 100, 47 and 41, respectively at the sn-1 position^6^. Yuzawa et al.^49^ showed the relative abundance of n-6 GLA, 18:2, and 18:1 fatty acid content in wild type *Synechocystis* sp. derived MGDG as 30%, 10%, and 5% respectively. However, the larger fatty acid chains at the sn-1 position have also been reported recently. The time of flight mass spectrometry (TOF–MS) analysis of the same cyanobacterial strain showed the predominance of 34:2 or 34:3 fatty acid chain at the sn-1 position^47^. Wada et. al.^48^ showed that the most abundant n-6 GLA moiety in *Synechocystis* sp. derived MGDG is subject to desaturation when the growth temperature was shifted from 38 °C to 22 °C. In contrast to the variable sn-1 fatty acid content, the sn-2 position was shown to be conserved mostly with 16: 0 (palmitic acid) fatty acid chain in *Synechocystis* sp. derived MGDG and was not affected with the shifts in temperature^6,48,49^.

The MGDG lipid molecule was isolated in a normal phase column using chloroform and acetone gradient eluent combinations. The column length to diameter ratio was ~ 10. Multiple samples were collected with each eluent combination for better purification. We confirmed the presence of MGDG molecule in fraction I using ^1^H NMR analysis (Fig. 3). Though fraction H, I, and J were eluted with the same mobile phase combination of 40% chloroform and 60% acetone, only fraction I contained the MGDG molecule. The peaks obtained for a fraction I identical with > 99% pure standard MGDG molecule suggests the high purity of MGDG molecule in that fraction. The diallylic methylene moiety obtained at δ 2.8 in the NMR analysis shown in Fig. 4 confirmed the polyunsaturation in the long fatty acid chain at the sn-1 position. The extracted MGDG molecule in fraction I was quantified using the HPLC–UV system. We prepared a calibration curve (Fig. 5) with known > 99% pure standard MGDG molecule to quantify the MGDG in each extracted fraction (~ 94.4 μg/ml). The peak obtained at retention time 6.24 min shown in Fig. 6, upper panel corresponds to the MGDG molecule in the fraction^43^. The large peak obtained at retention time 2.36 min is the lipid pigments eluted initially by the chloroform. The peak at 7.23 min might be due to the presence of steryl glucosides present as a contaminant in the fraction^43^. Glucosides do not have any known effects on the HER2-positive and triple-negative breast cancer cells. The peaks for pigments and glucosides were also observed in the standard > 99% pure standard MGDG chromatogram (Fig. 6, lower panel). So, the contaminant should not have any effect in this study during the drug formulation used for cytotoxicity analysis. However, the GGL molecules are detected with high quality and precision using the evaporative light scattering detectors (ELSD) providing with sharp and clear peaks in the chromatogram^43,58,59^.

We investigated the in vitro anti-proliferation efficacy of cyanobacteria derived MGDG molecule in comparison with MGDG molecule from plant source on the HER2-positive and triple-negative breast cancer cell lines. We observed significant differences in the % of cancer cell death using MGDG extracted from *Synechocystis* sp. *versus* a commercially available MGDG standard. MGDG extracted from *Synechocystis* sp. induced apoptosis in (70 ± 8) % of BT-474 and (58 ± 5) % of MDA-MB-231 cells at 60 and 200 ng/ml, respectively, while < 15% cell death was observed in fibroblasts at 100 ng/ml (Fig. 7). While the precise mechanism by which each molecule works about their cell-killing effect is unknown, it is possible that the presence of polyunsaturated GLA in the sn-1 position and palmitic acid in the sn-2 position of MGDG from *Synechocystis* sp. resulted in the mitochondrial depolarization, cytochrome c release, DNA fragmentation and generation of free radicals causing specific cell death response in breast cancer cells compared to normal cells^60–64^. GLA has been shown to exhibit anti-proliferative activities specifically in a variety of cancer cell lines both in vitro and in vivo. GLA inhibited the cell growth of four human neuroblastoma cell lines (GOTO, SK-N-DZ, NKP, and NCG) in vitro^65^, three human glioma cell lines (MOG, U87, U373) and a rodent glioma cell line (C6) in vitro and a rat C6 glioma model in vivo^66^. Glioma regression and apoptosis had been reported using both C18 and C20 fatty acids of the n-6 and n-3 series GLA along with the preservation of normal neural tissue and vasculature in adjacent brain^66^. GLA at a concentration of 150 μM inhibited Walker 256 cancer cell growth both in vitro and in vivo causing decrease in mitochondrial membrane potential, and increase in cytochrome c release, caspase activation, and DNA fragmentation^61^. The mitochondrial apoptosis pathway was likely induced by an increase in reactive oxygen species (ROS), lipid peroxide production, ATP generation and the deposition of large amounts of triacylglycerol in the form of lipid droplets^61,63^. A diet containing 5.5% GLA caused 45% decrease in Walker 256 tumor growth in vivo by reducing mitochondrial metabolic activity^60^. More interestingly, in vitro, in vivo and clinical study data showed that GLA has selective anti-proliferative actions in cancer cells with little or no side effects on normal cell growth. Polyunsaturated fatty acids including GLA incubated with human breast, lung, and prostate cancer cells suppressed the cancer cell growth exhibiting no adverse effects on normal human fibroblasts or normal animal cell lines^67^. Intraarterial injection of a lithium salt derivative of GLA demonstrated its ability to selectively suppress angiogenesis^62^. These reports, together with our data increases lead to the conclusion that MGDG from *Synechocystis* sp. is a promising cancer therapeutic agent with high selectivity of cancer cell growth inhibition leading to apoptosis and a decrease in cancer development. Also, saturated fatty acid, palmitic acid at the sn-2 position of MGDG from *Synechocystis* sp. plays a significant role in the elevation of calcium flux, endoplasmic reticulum stress, caspase-3, and caspase-9 activity, and thus inducing apoptosis which is in good agreement with previous reports^68–70^. Treatment of mouse 3T3-L1 and rat primary preadipocytes with palmitic acid induced multiple cell signaling pathways, endoplasmic reticulum stress responses, and cell cycle arrest leading to apoptosis^68^. Palmitic acid induced oxidative stress and DNA damage in rodent-derived insulin-secreting cell line RINm5F and primary human fibroblasts^70^. Spinach MGDG molecule in combination with gemcitabine in vitro^57^ and radiation in vivo^71^ showed enhanced suppression of MIAPaCa-2, PANC-1, and BxPC-3 pancreatic cancer cell lines compared to MGDG treatment alone. With the in vitro spinach MGDG treatment alone the IC_50_ values for the mentioned pancreatic cell lines ranged from 18 to 25 μM^71,72^. MGDG molecule extracted from the spinach has been shown to inhibit human replicative and repair DNA polymerase enzymes with the IC_50_ values ranging from 10 to 200 μM^57^. These results illustrate the specificity of the MGDG extracted from *Synechocystis* sp. PCC 6803 to breast cancer cells by caspase-dependent apoptotic pathway (Fig. 8).

## Conclusion

Fatty acids are of great interest as natural anti-proliferative factors because of their selective inhibition of cancer cells. The structure and efficacy of fatty acid molecules are variable within and across the sources. We investigated the cancer cell inhibition efficacy of plant and cyanobacterial derived MGDG molecule. The marine cyanobacteria *Synechocystis* sp. derived MGDG molecule predominantly rich in n-6 GLA was isolated from the total lipid extract in this study. The molecule was isolated in a hand-packed low-cost normal phase silica column with the gradient elution method using chloroform and acetone as the mobile phase. We confirmed the presence of MGDG molecule in the fraction eluted with 40% chloroform and 60% acetone eluent combination. The NMR analysis confirmed the high purity of the isolated MGDG molecule from the cyanobacterial total lipid extract. The isolated MGDG molecule was quantified by the HPLC–UV system. The anti-proliferative activity of the cyanobacterial MGDG was tested against the MDA-MB-231 breast cancer cell line. The in vitro cytotoxicity study showed that MGDG extracted from *Synechocystis* sp. PCC 6803 potently inhibited (70 ± 8) % and (58 ± 5) % of BT-474 and MDA-MB-231 cells, respectively using 60 and 200 ng/ml of the MGDG. The IC_50_s of MGDG extracted from *Synechocystis* sp. are (27.2 ± 7.6) and (150 ± 70) ng/ml in BT-474 and MDA-MB-231 cells, respectively. In contrast, plant extract n-3 ALA-rich MGDG did not have any cytotoxic effect in the concentration range of 0–200 ng/ml. It did not show any cytotoxic effects in human dermal fibroblasts normal cell controls. Our results support these findings showing that the cyanobacteria derived MGDG induces caspase-dependent apoptosis pathway to inhibit the HER2-positive BT-474 and triple-negative MDA-MB-231 breast cancer cell growth. Further studies involving additional mechansisms of actions, intracellular uptake, and robust screening of additional cancer cell lines will confirm the potential novel therapeutic efficacy of cyanobacteria derived MGDG molecule for a low dosage selective treatment of breast cancer cells.

## Supplementary Information

Supplementary Information

Supplementary Video
