# Supplementary material for: Isolation and purification of glycoglycerolipids to induce apoptosis in breast cancer cells
Source: Sci Rep. 2021 Jan 14;11:1298. doi: 10.1038/s41598-020-80484-x (PMC7809038; doi:10.1038/s41598-020-80484-x)
Supplement: Supplementary file 1 — Supplementary Information [file 41598_2020_80484_MOESM1_ESM.pdf]

Supplementary Information

Isolation and Purification of Glycoglycerolipids to Induce Apoptosis in Breast Cancer Cells

Muhammad Raisul Abedin and Sutapa Barua [1]

Department of Chemical and Biochemical Engineering

Missouri University of Science and Technology

Rolla, MO 65409

[1] To whom correspondence should be addressed. Email: baruas@mst.edu. Department of Chemical & Biochemical Engineering, 110 Bertelsmeyer Hall, 1101 N. State Street, Rolla, MO 65409-1230

(a)

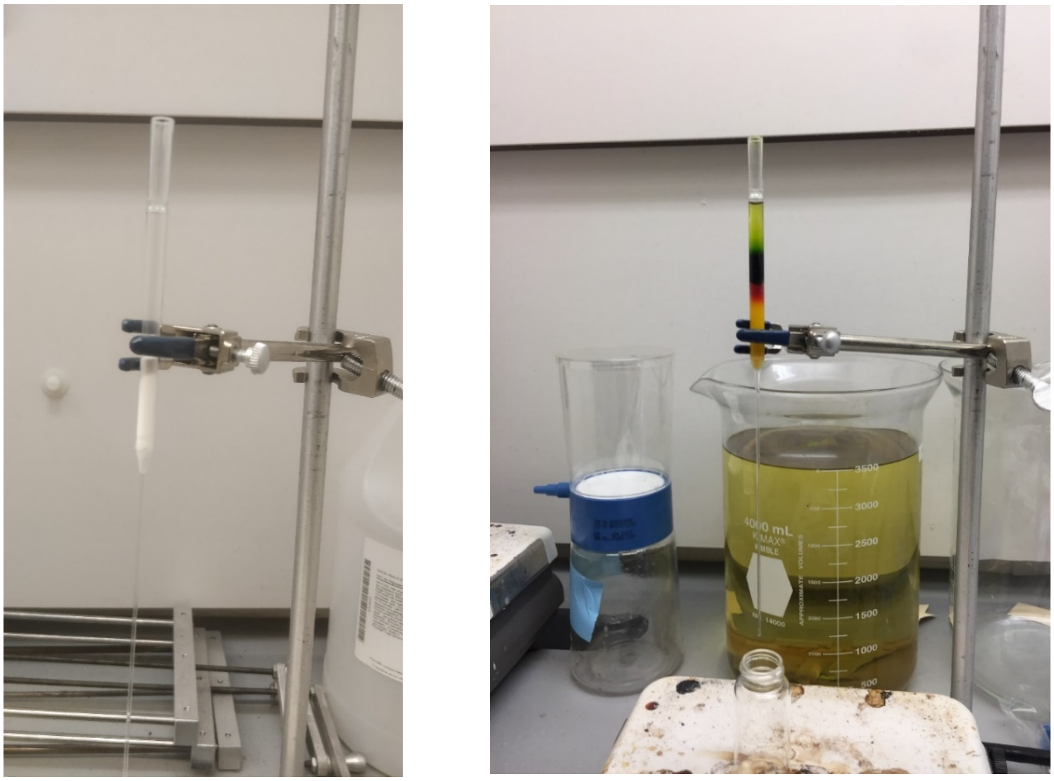

(b)

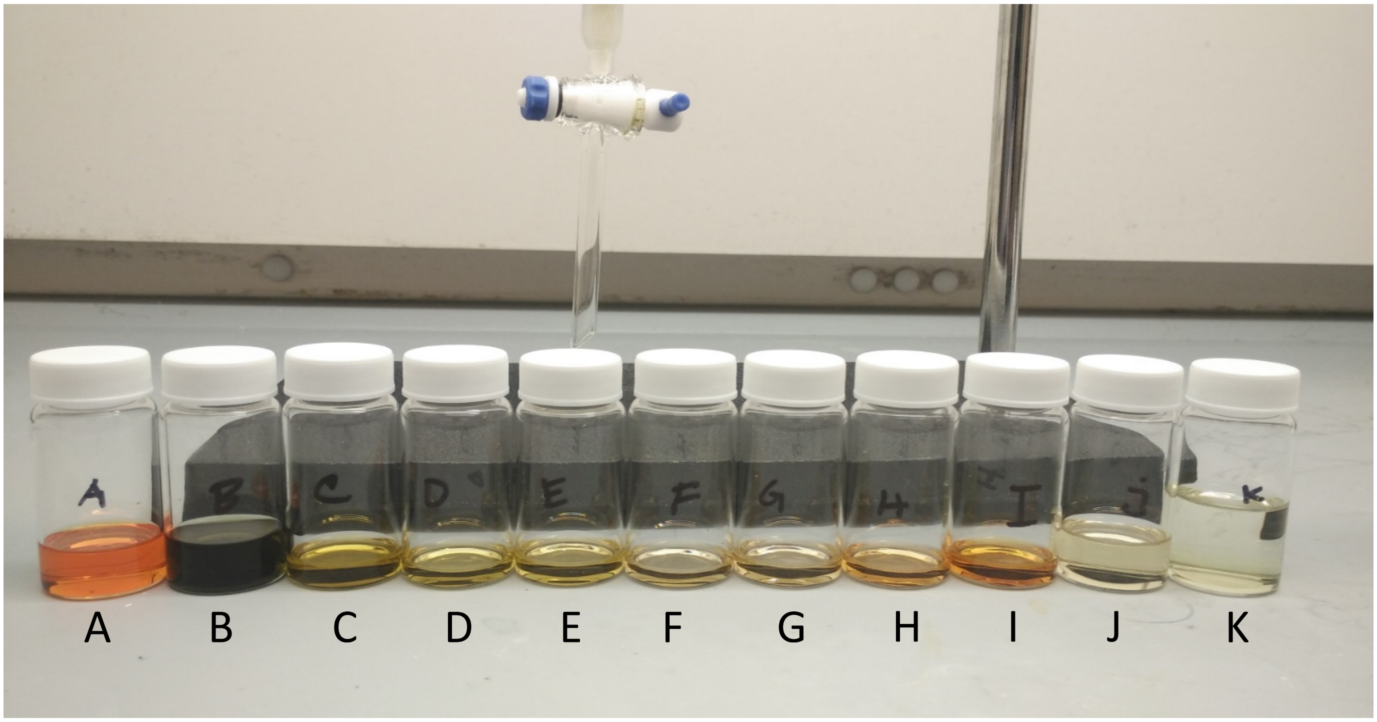

SI Figure 1: Fractionation of total lipid using column chromatography technique.

(a) Fractionation of total lipid in a handmade column. Silica gel (10-40 µm) was used as a stationary phase. Chloroform and acetone were used as the mobile phase.

(b) The fractionated samples were collected separately by observing the color difference of individual lipid class. Eluent 1: fraction A, eluent 2: fraction B, eluent 3: fraction C, D, E, F, G, eluent 4: fraction H, I, J, eluent 5: fraction K.

SI Table 1: Chromatographic fractionation method of total lipid, A-Chloroform; B-Acetone

**Eluent**	**A (%)**	**B (%)**	**Volume (ml)**
1	100	0	10
2	90	10	10
3	75	25	10
4	40	60	10
5	0	100	10

SI Table 2: HPLC column preparation and conditions for detection and quantification of MGDG

**Column**	LiChrospher 100 Diol 25-4, 5 μm (Normal Phase Column)
**Detector**	UV (240 nm)
**Mobile phase**	Solution A: Chloroform, Solution B: Methanol/water (95:5 v/v)
**Flow rate**	1 ml/min
**Injection volume**	25 μl
**Column temperature**	RT

SI Table 3: HPLC elution method for detection and quantification of MGDG

Time (min)	A (%)	B (%)
0	99	1
15	75	25
20	10	90
25	10	90
30	99	1

SI Table 4: Corresponding peak areas of increasing concentration of standard MGDG

Concentration (μg/ml)	Peak Area (mAU.s)
0	0
5	24.57
8	47.26
10	66.28
50	596.63
100	1610.78
150	2505.67

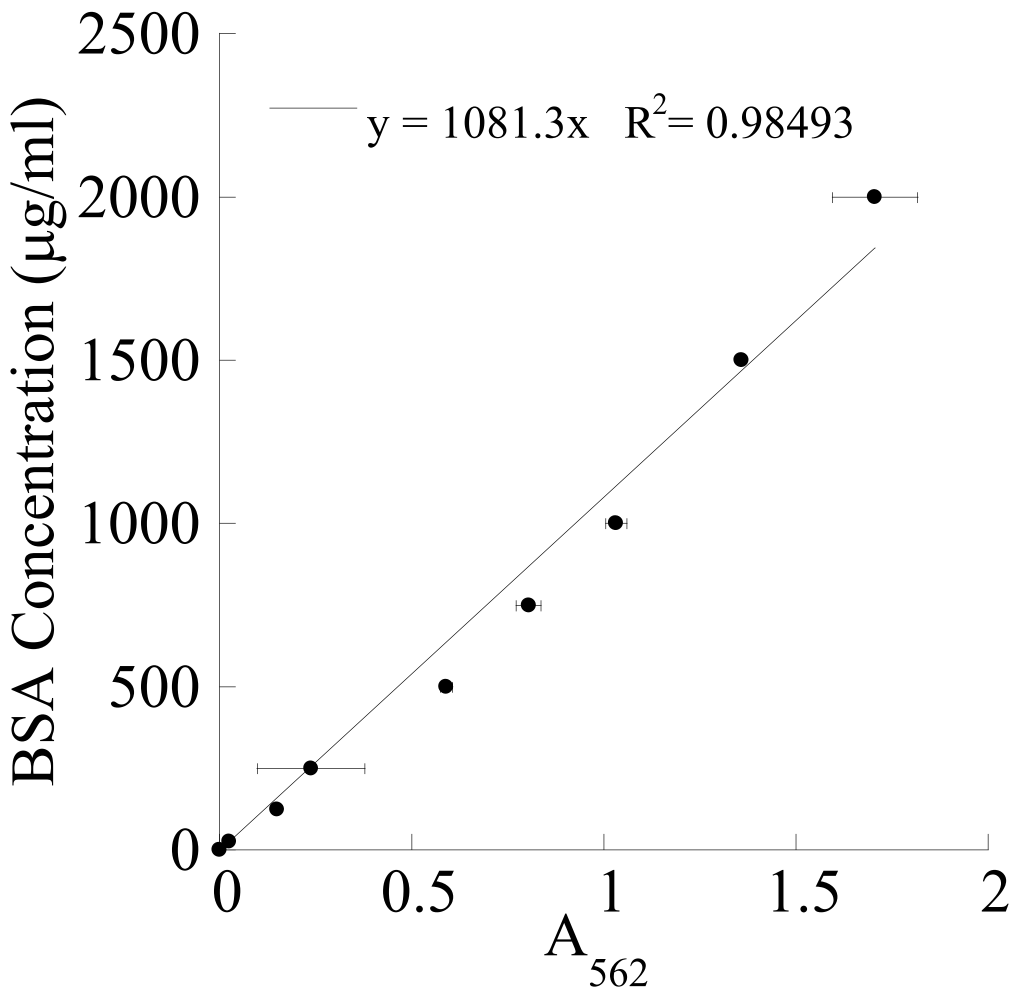

SI Figure 2: Calibration curve of BSA protein for quantification of protein in cell lysates for Western blot experiments. The calibration curve was obtained using a BCA protein quantification assay at 562 nm absorbance values.

Western blot analysis

Western blot analysis was performed to verify the active caspase-3 expression of the standard and *Synechocystis* sp. extracted MGDG treated BT-474 and MDA-MB-231 cells. Approximately, 10^6 cells were seeded in 25 cm^2 cell culture flasks and treated with 100 ng/ml standard MGDG and *Synechocystis* sp. extracted MGDG. After 72 h of incubation, the cells were trypsinized for total protein extraction using the radioimmunoprecipitation assay (RIPA) lysis buffer. The total protein was quantified using the BCA calibration curve **(SI Figure 2)**. For SDS-PAGE, the samples were prepared in 2X Laemmli loading buffer. The samples were denatured at 86°C for 2-3 min and loaded on the 16% Novex Tris-Glycine gels (Invitrogen). The gel was run in tris-glycine-SDS running buffer at constant 200 V for 30-40 min. The separated protein bands on the gel were transferred to the nitrocellulose membrane using the Power Blotter (Invitrogen). The membrane blot was rinsed briefly with tris buffered saline including 1% tween-20 (TBST) for 2-3 min and was blocked with 3% bovine serum albumin (BSA) protein (Fisher Scientific) at room temperature for 5 h. After blocking, the membrane was incubated overnight in the primary antibody caspase-3 (ThermoFisher, Cat. 43-7800) and actin (ThermoFisher Cat. MA5-11869) at 4°C with the dilution factor of 1:500 and 1:3000, respectively. The membrane was washed 3-5 times with TBST buffer for 5 min each followed by the incubation with anti-mouse horseradish peroxidase (HRP) conjugated secondary antibody (ThermoFisher Cat. A27025). The membrane blot was incubated in secondary the antibody for 1 h with the dilution factor of 1:10,000 followed by washing with TBST buffer. Finally, the blot was incubated in electro-chemiluminescent reagent (Super Signal West Dura, ThermoFisher, and Cat. 34075) for 5 min and was imaged using a Bio-Rad gel imaging system.

(a)

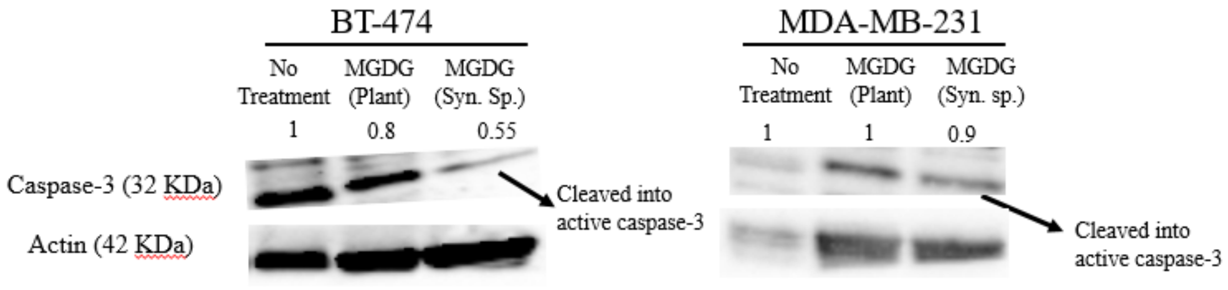
BT-474
No Treatment
MGDG (Plant)
MGDG (Syn. Sp.)
1
0.8
0.55
Caspase-3 (32 KDa)
Actin (42 KDa)
Cleaved into active caspase-3
MDA-MB-231
No Treatment
MGDG (Plant)
MGDG (Syn. sp.)
1
1
0.9
Cleaved into active caspase-3

(b)

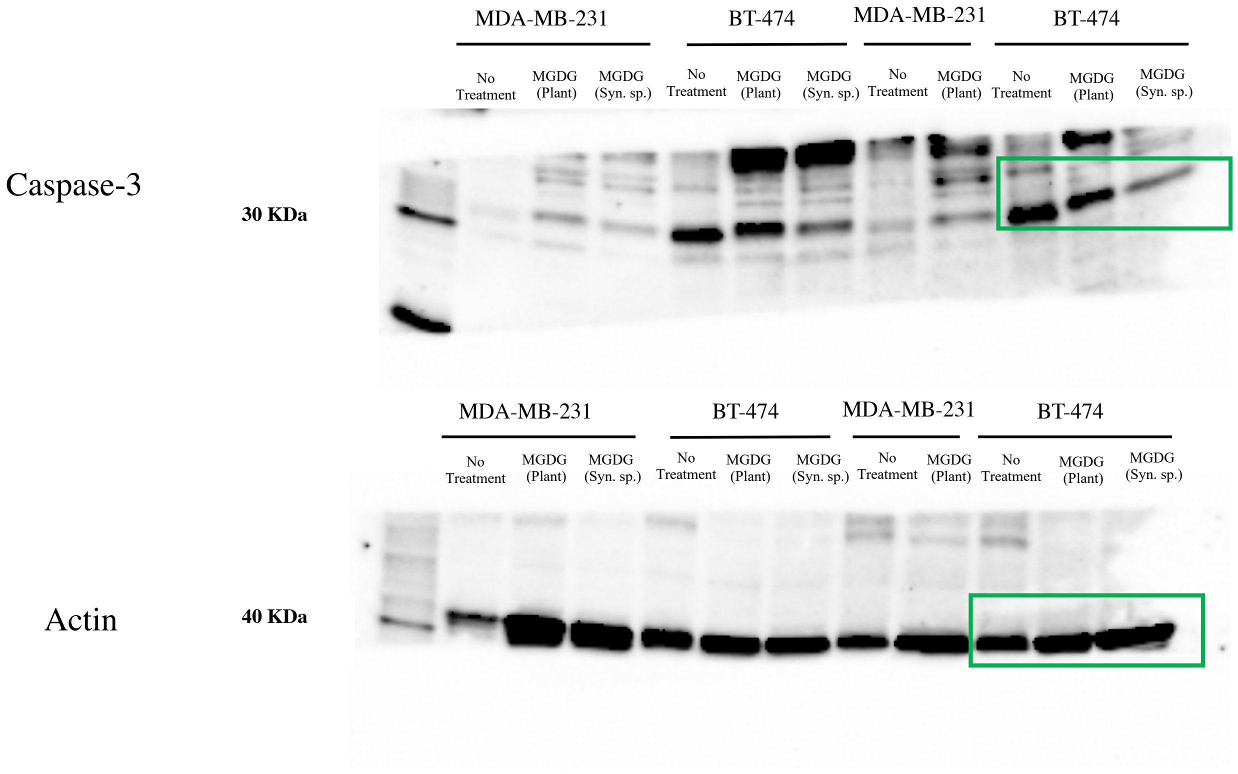
MDA-MB-231
BT-474
MDA-MB-231
BT-474
No Treatment
MGDG (Plant)
MGDG (Syn. sp.)
Caspase-3
30 KDa
Actin
40 KDa

(c)

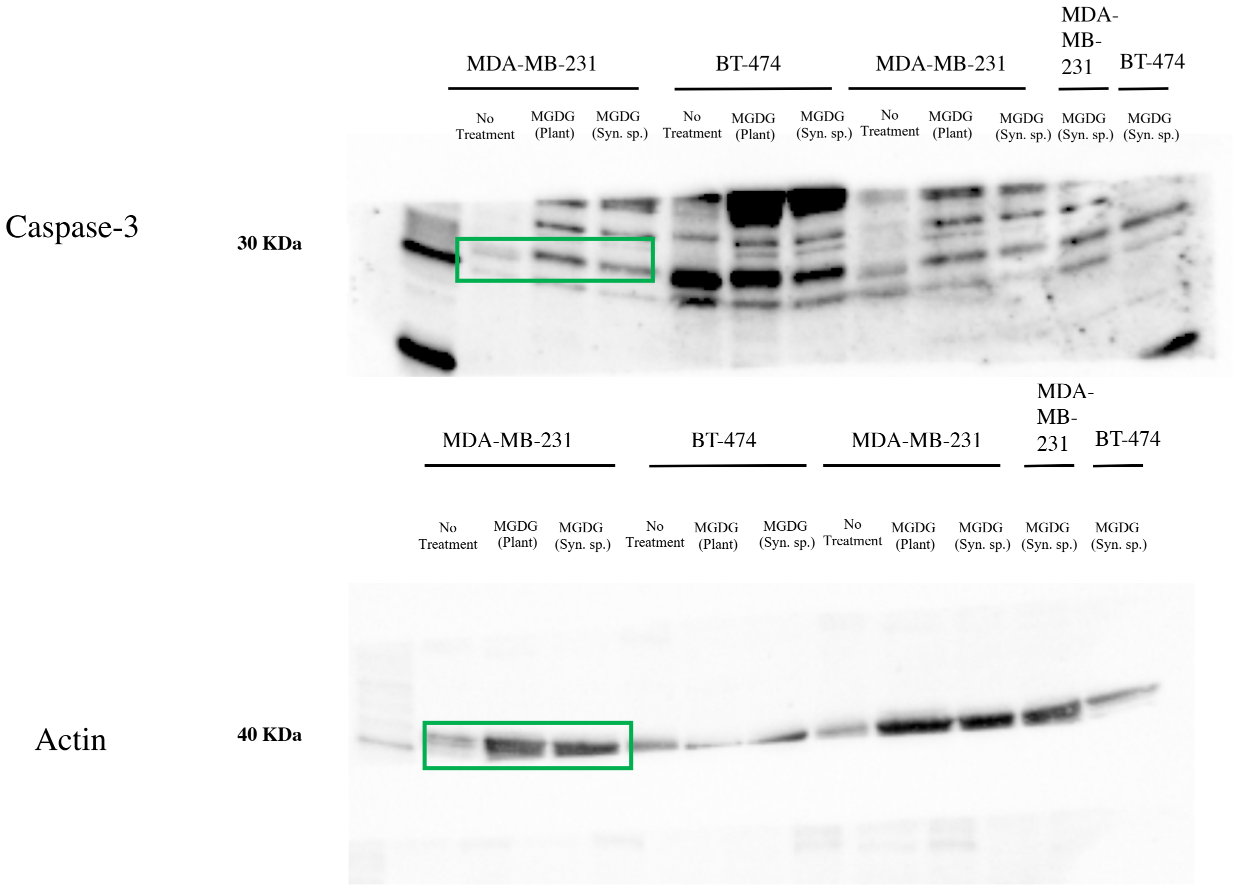

SI Figure 3 (a): The Western blot assay confirming the reduced full-length caspase-3 protein expressions in BT-474 (left panel) and MDA-MB-231 (right panel) cell lines treated with MGDG extracted from *Synechocystis* sp. compared to untreated and MGDG standard treated samples. Caspase-3 (32 kDa) is cleaved into lower molecular weight (17kDa) active caspase-3/7 that is shown by fluorescence microscopic images and flow cytometry quantification in Figure 8 of the main texts. **(b)** and **(c)** The full blot of Western blot images are shown. The green rectangular outlines represent the selected areas of the gel displayed in **(a)**.

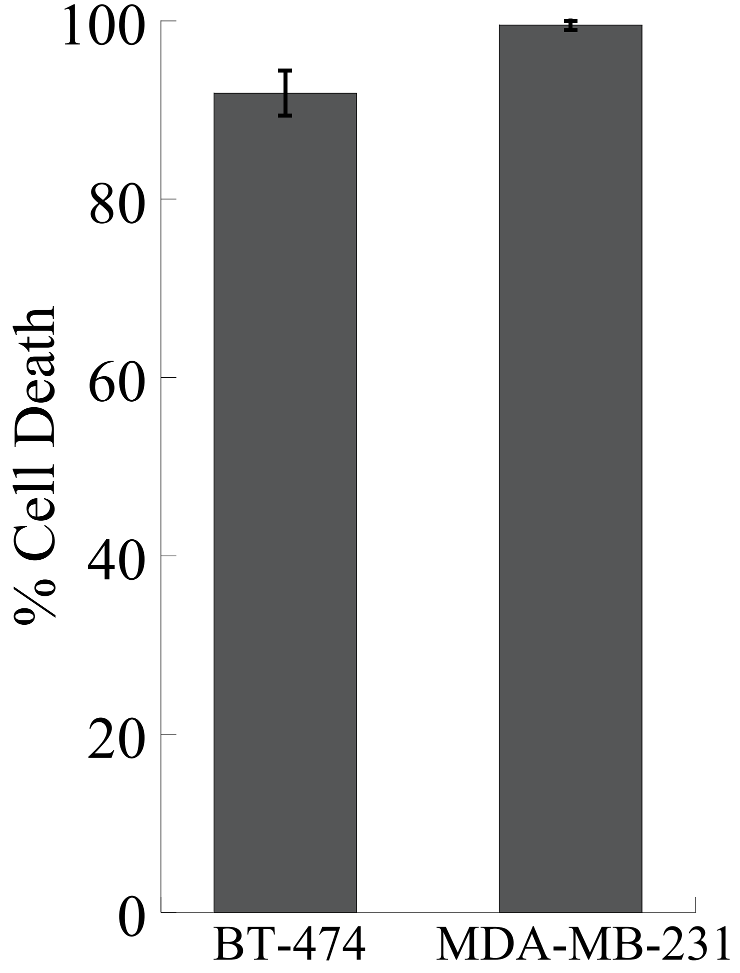

SI Figure 4: Dead control cells induced by saponin was used in MTT assay

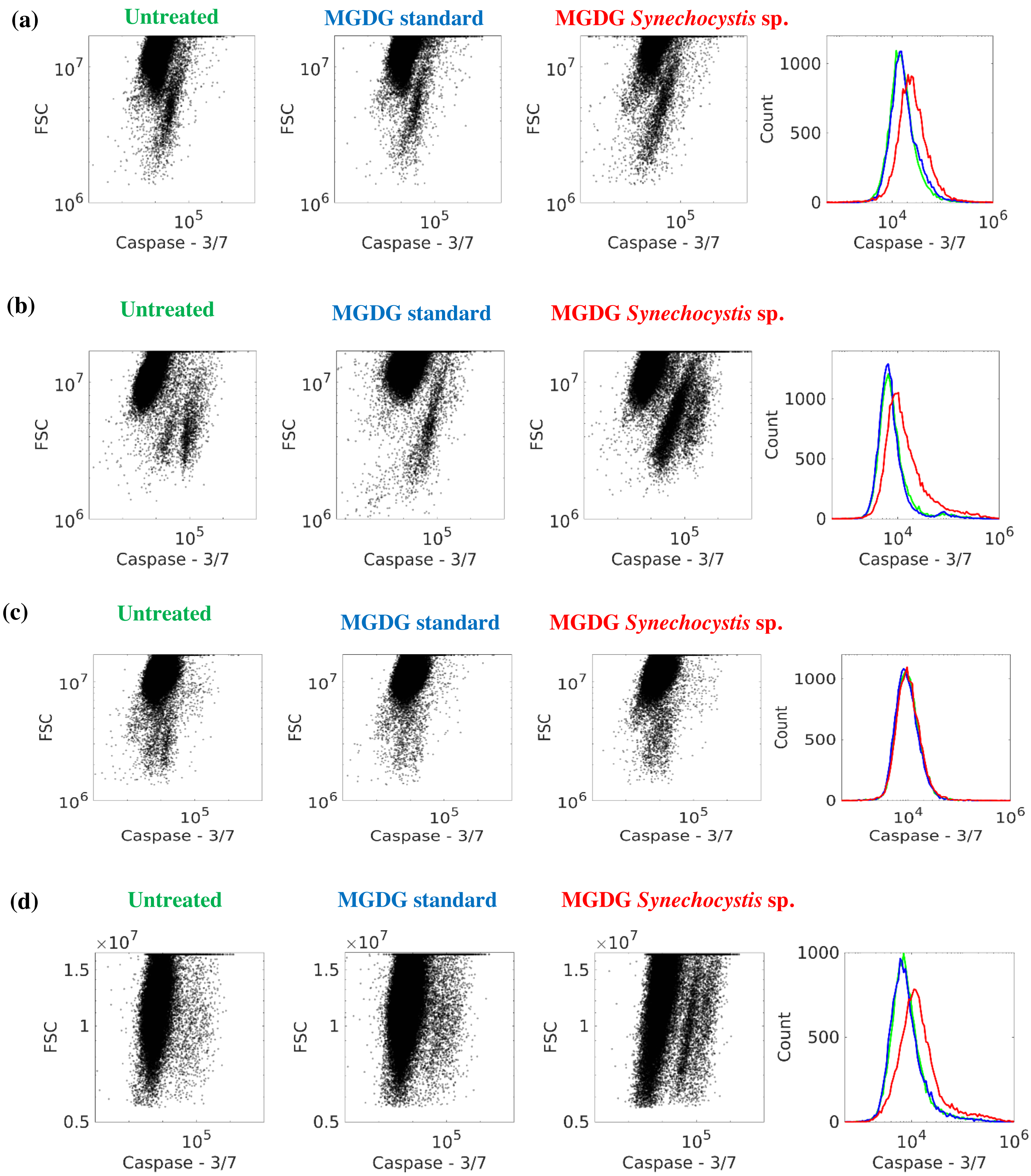
(a)
Untreated
MGDG standard
MGDG Synechocystis sp.
FSC
Count
Caspase - 3/7
(b)
Untreated
MGDG standard
MGDG Synechocystis sp.
FSC
Count
Caspase - 3/7
(c)
Untreated
MGDG standard
MGDG Synechocystis sp.
FSC
Count
Caspase - 3/7
(d)
Untreated
MGDG standard
MGDG Synechocystis sp.
FSC
Count
Caspase - 3/7

SI Figure 5. Quantification of apoptosis assay. For quantification of apoptosis induction the BT-474 and MDA-MB-231 cells were treated with standard plant extract MGDG and *Synechocystis* sp. extracted MGDG with 100 ng/ml dose. The cleaved caspase-3/7 fluorescence intensity data were collected using flow cytometry. The scatter plots were generated for each treatment showing cleaved caspase-3/7 fluorescence data in x-axis and forward scattering (FSC) values in y-axis. A combined histogram was also generated for each treatment showing cleaved caspase-3//7 fluorescence intensity distribution in cell population. The green, blue and red lines represent the histogram data for untreated, MGDG standard treated and *Synechocystis* sp. extracted MGDG treated samples. The scatter plots and combined histograms were generated for treatments in BT-474 cell line after **(a)** 48 h and **(b)** 72 h and in MDA-MB-231 cell line after **(c)** 48 h and **(d)** 72 h. In BT-474 cell line, the mean cleaved caspase-3/7 fluorescence intensity was shifted by 1.7±0.04 ($p<0.01$) and 2.34±0.44 ($p<0.01$) fold in *Synechocystis* sp. extracted MGDG treated cells compared to untreated control cells after 48 h and 72 h of treatment, respectively. In MDA-MB-231 cell line, the shift in mean fluorescence intensity was 0.95±0.01 and 2.1±0.03 fold ($p<0.01$) in *Synechocystis* sp. treated cells after 48 h and 72 h, respectively compared to untreated control cells.
